# The effect of *APOE*4 on Alzheimer’s plasma biomarkers among Mexican Americans in the HABS-HD cohort

**DOI:** 10.1186/s13195-025-01845-0

**Published:** 2025-09-30

**Authors:** J. A. Contreras, N. E. Ortega, K. Espejo, V. Aslanyan, J. Pa, The HABS-HD Study Team, Sid E. O’Bryan, Kristine Yaffe, Arthur Toga, Robert Rissman, Leigh Johnson, Meredith Braskie, Kevin King, James R. Hall, Melissa Petersen, Raymond Palmer, Robert Barber, Yonggang Shi, Fan Zhang, Rajesh Nandy, Roderick McColl, David Mason, Bradley Christian, Nicole Phillips, Stephanie Large, Joe Lee, Badri Vardarajan, Mónica Rivera Mindt, Amrita Cheema, Lisa Barnes, Mark Mapstone, Annie Cohen, Amy Kind, Ozioma Okonkwo, Raul Vintimilla, Zhengyang Zhou, Michael Donohue, Matthew Borzage, Michelle Mielke, Beau Ances, Ganesh Babulal, Jorge Llibre-Guerra, Carl Hill, Rocky Vig

**Affiliations:** 1 Department of Neurosciences, Alzheimer’s Disease Cooperative Study (ADCS), University of California, 9500 Gilman Drive, MC 0949, La Jolla, San Diego, CA 92093-0949 USA; 2 Department of Population and Public Health Sciences, Keck School of Medicine, University of Southern California, Los Angeles, CA USA

## Abstract

**Background and objectives:**

The relationship between *APOE*4 status and plasma biomarkers previously shown to be related to Alzheimer’s risk have not been carefully examined among Mexican Americans. This research is needed to elucidate disparities within the Alzheimer’s field by evaluating key genetic factors in an underrepresented population. The present study deepens our understanding of the interaction between biological and genetic factors for these populations with greater incidence of Alzheimer’s disease (AD) and helps address whether *APOE*4 confers similar risk of AD in Mexican Americans as previously reported in Non-Hispanic Whites.

**Methods:**

Cross-sectional data consisting of 792 Mexican American and 785 Non-Hispanic White participants from the Health & Aging Brain Study – Health Disparities (HABS-HD) with available plasma biomarkers and *APOE*4 genotype profiles were included in the present study. Linear regression models were used to test our hypotheses. *APOE*4—Race/ethnicity interaction term tested whether the biomarker levels differed between ethnic groups. Analyses were adjusted for age, gender, and education. Further analyses explored whether biomarker levels differed by *APOE*4 carrier status within racial/ethnic groups.

**Results:**

Among 1577 participants (59.5% women; mean age 66.4 ± 8.74 years), significant differences were observed across race/ethnic and *APOE*4 groups. Mexican Americans were younger (*p* < 0.001), had a higher proportion of women (*p* = 0.001), fewer years of education (*p* < 0.001), and lower MMSE scores (*p* < 0.001). Biomarker differences between Mexican Americans and Non-Hispanic Whites included variations in Aβ42/Aβ40 (*p* = 0.03) and p-tau181 (*p* < 0.001), but not in total tau, TNF-α, or NfL levels (all *p* > 0.12). Race/ethnicity—*APOE*4 interactions were significant for Aβ42/Αβ40, p-tau181, and total tau (all *p* < 0.05) but not for NfL or TNF-α. *APOE*4 associations with Aβ42/Aβ40 and p-tau181 were significant in NH White participants (all *p* < 0.001) but not among Mexican Americans.

**Conclusion:**

These findings will significantly contribute to understanding potential differences in the role of *APOE*4 and AD plasma biomarkers among Mexican Americans. This research has the potential to enhance preventive care and early diagnosis for populations with a higher incidence of AD.

**Supplementary Information:**

The online version contains supplementary material available at 10.1186/s13195-025-01845-0.

## Introduction

Alzheimer’s Disease (AD) is the most common form of dementia among the elderly, affecting an estimated five million individuals in the United States. By 2050 that number is expected to rise to well over 10 million Americans [1]. Adding to this, the rate of AD is higher among Hispanics/Latinos (65% of which are Mexican American [MA]), compared to Whites [1]. Most research on AD genetic risk factors, such as *APOE*4, has highlighted their significant associations with AD biomarkers and contributing to the development of mechanistic models that inform AD drug development [2]. For instance, individuals carrying *APOE*4 allele often exhibit altered levels of certain plasma biomarkers associated with AD, particularly amyloid-beta (Aβ) peptides [3], with higher doses of *APOE*4 generally correlating with lower plasma Αβ42/Aβ40 levels, indicating increased amyloid plaque deposition in the brain [4]. Similarly, *APOE*4 is strongly associated with increased levels of tau plasma biomarkers [5] with the proposed mechanism that the presence of *APOE*4 may influence tau protein metabolism, leading in increased phosphorylation into the bloodstream [6, 7]. Yet, unfortunately, most of the research investigating *APOE*4 has been conducted in predominantly Non-Hispanic White cohorts. The resulting knowledge declares *APOE4* as the major genetic risk factor for developing late-onset AD [8], but this association may not generalize to other diverse ethnic populations.

For instance, research studies conducted on African American cohorts have shown that the relative effect of *APOE*4 may not be meaningful in ethnically diverse cohorts. Powell and colleagues acknowledged that, while African Americans had a higher prevalence of the *APOE*4 allele, White *APOE*4 carriers had a greater risk of dementia [9]. Specifically, they found that onset of dementia is earlier in African Americans regardless of *APOE*4 status. This finding helps shed light on the fact that genetic risk factors are not as ubiquitous as we had previously thought. Recent studies report that Mexican American present with earlier age of onset for cognitive decline and neurodegeneration compared to Non-Hispanic White participants [10, 11], yet tend to exhibit lower rates of amyloid positivity and lower prevalence of the *APOE4* genotype [12]. Studies examining whether *APOE*4 serves as a risk factor for AD among MA are limited.

*APOE* is a lipid-trafficking protein that impacts inflammation and has well described disease-associated (e4) alleles. Lipids play essential roles in immune regulation as a major component of cell membranes and as ligands for various immune receptors to mediate immune cell activation and phagocytosis. Studies have found that a single copy of *APOE*4 is associated with marked augmentation of the human innate immune response [13], supporting previous studies showing that *APOE*4 carriers have heightened systemic inflammation after acute conditions and injuries [14]. Despite their importance and possible contribution to aging and AD, studies on *APOE*4 and the link to inflammatory biomarkers in underrepresented groups such as Mexican Americans are scarce.

Using the Health & Aging Brain Study—Health Disparities (HABS-HD) cohort, the present study investigated whether *APOE*4 is associated with AD biomarkers, including Aβ, phosphorylated and total-tau, and inflammation, in Mexican Americans as has been seen previously in Non-Hispanic Whites. AD biomarker levels across *APOE*4 carrier status and racial/ethnic groups were compared between groups.

## Methods and methods

### Study design and clinical cohort

Cross-sectional data was analyzed from 792 MA and 785 Non-Hispanic White participants in the Health & Aging Brain Study – Health Disparities (HABS-HD; formally the Health & Aging Brain study among Latino Elders, HABLE study) who had available plasma biomarkers and *APOE* genotype data (see supplemental Fig. 1). The HABS-HD study is an ongoing, longitudinal, community-based project examining health disparities in mild cognitively impaired and AD among Hispanics identifying as Mexican Americans as compared to non-Hispanic white participants [15]. HABS-HD methods have been published elsewhere [15] and are briefly outlined below. Inclusion criteria for the study includes (1) self-identifying as either Mexican American (MA) or non-Hispanic white (NH White), (2) willingness to provide blood samples, (3) capable of undergoing neuroimaging studies, (4) age 50 and above, and (5) fluent in English or Spanish. Exclusion criteria includes (1) Type 1 diabetes, (2) presence of active infection, (3) current/recent (12 month) cancer (other than skin cancer), (4) current severe mental illness that could impact cognition (other than depression), (5) recent (12 months) traumatic brain injury with loss of consciousness, (6) current/recent alcohol/substance abuse, and (7) active severe medical condition that could impact cognition (e.g., end stage renal failure, chronic heart failure, chronic obstructive pulmonary disease). All aspects of the study protocol have been conducted in Spanish or English. The HABS-HD study is conducted under IRB approved protocols and each participant (or his/her legal representative) has given written informed consent. All HABS-HD data is available to the scientific community through the UNTHSC Institute for Translational Research (ITR) website.

### Diagnostic classification

APOE4 genotyping was performed using commercially available TaqMan Genotyping Kits for rs429158 and rs7412 using TaqMan GTXpress Master Mix (ThermoFisher). Target amplification and detection was performed using the 7,500 RealTime PCR System (Applied Biosystems). Genotypes were called according to combined of allele amplification results at the two SNPs as follows (rs429358, rs7412): *APOE*—2/2; 2/3; 2/4; 3/3; 3/4; 4/4. Positive controls (individuals of known, independently typed APOE genotypes) and negative controls were included on all runs. *APOE* genotype frequencies were confirmed to be in Hardy–Weinberg equilibrium [12].

APOE genotype was dichotomized into ε4 carriers (APOE 3/4 or 4/4) and non-carriers (APOE 2/2, 2/3, or 3/3) to examine the effect of the ε4 allele on cognitive and pathological outcomes. This approach was selected to preserve statistical power, particularly in analyses involving Mexican American participants, among whom ε4 allele frequency is relatively lower.

### Blood collection and processing procedures

Fasting blood samples were collected, processed, and stored per previously published international guidelines (O’Bryant et al., 2014). Assay preparation was completed using custom automated StarPlus system from Hamilton Robotics (Reno, NA). Plasma markers of amyloid (Aβ 40, Aβ 40), tau (t-tau), phosphorylated tau (p-tau), neurodegeneration (NfL), and inflammatory plasma marker (tumor necrosis factor-α) were assayed using the ultra-sensitive Simoa (single molecule array technology) on the HD-X platform (Quanterix.com, Billerica, MA). Coefficients of variation for all assays were of ≤ 5%. Complete details can be found here ( [15–18]). Lower Aβ42 values is an indicator of a greater presence of Aβ in the brain, which is contrary to the other plasma markers in which higher levels indicate greater AD risk.

### Neuropsychological assessment

An interview is conducted as part of the HABS-HD protocol, which includes neuropsychological testing used in this assessment: Mini Mental Status Exam (MMSE) [10, 19].

### Statistical analysis

All analyses were conducted in R (version 4.4.1). Demographics, biomarker levels, and cognition markers were compared between ethnic groups using ANOVA for continuous variables and χ^2^ tests for categorical variables. Race/ethnicity was modeled as a categorical variable using dummy coding. The included racial/ethnic groups were non-Hispanic White and Mexican Americans. Within supplemental material, we included pairwise comparisons using Tukey’s Honestly Significant Difference (HSD) where NHW non-carriers were used as the reference group (supplemental Fig. 2).

To test our main hypotheses, we used linear regression models, adjusting for age, gender, and education. The multiplicative APOE4 × race/ethnicity interaction term was included to examine whether biomarker levels differed between ethnic groups. Regression assumptions were checked through statistical testing and visual inspection. Post-hoc analyses were conducted to identify influential observations, and sensitivity analyses were repeated after removing these observations.

Additional analyses explored differences in biomarker levels by APOE4 carrier status within ethnic groups, using similarly adjusted linear regression models (again covarying for age, gender and education). Given the expected correlation among selected biomarkers (due to shared pathological signatures), *p*-values for comparisons were corrected using the Benjamini–Hochberg procedure to control for false discovery rate. To examine the potential influence of baseline cognitive status, we included Mini-Mental State Examination (MMSE) scores as an additional covariate in all regression models.

## Results

### Demographics and the study participants

One thousand five hundred seventy-seven participants with complete biomarker profiles were included in the study. 59.5% of participants were women with a mean (SD) age of 66.4 (8.74). When stratified by race/ethnicity and carrier status, significant group differences were observed for age, gender, education, and cognition: Mexican Americans were generally younger (*p* < 0.001), had a larger proportion of women (*p* = 0.001), had less years of education (*p* < 0.001) and lower MMSE scores (*p* < 0.001). Biomarker differences were mixed: while there were differences in Aβ42/Aβ40 levels, primarily driven by *APOE*4 status (*p* = 0.03), and in p-tau181 levels, driven by race/ethnicity groups (*p* < 0.001), No differences were observed in total tau (*p* = 0.34), TNF-α (*p* = 0.28), and NfL levels (*p* = 0.12) (Table 1).

**Table 1:** Participant demographic, biomarker, and cognitive characteristics

	**Non-Hispanic Whites**		**Mexican Americans**		***p*-value**
	**Non-carrier**	**Carrier**	**Non-carrier**	**Carrier**	
	**(*N* = 555)**	**(*N* = 230)**	**(*N* = 647)**	**(*N* = 145)**	
**Age, years**	69.3 (8.67)	68.4 (8.67)	63.8 (7.86)	63.4 (8.56)	< 0.001
**Gender**					0.001
Men	250 (45.0%)	107 (46.5%)	233 (36.0%)	48 (33.1%)	
Women	305 (55.0%)	123 (53.5%)	414 (64.0%)	97 (66.9%)	
**Education, years**	15.6 (2.63)	15.6 (2.51)	9.64 (4.61)	10.0 (4.77)	< 0.001
**Aβ42/Aβ40**	0.0518 (0.0124)	0.0469 (0.0122)	0.0503 (0.0141)	0.0489 (0.0114)	0.03
**total-tau (pg/mL)**	2.17 (0.880)	2.23 (1.10)	2.27 (0.885)	2.12 (0.668)	0.34
**p-tau181 (pg/mL)**	2.24 (1.52)	2.81 (1.49)	1.90 (1.21)	2.04 (1.19)	< 0.001
**TNF-α (pg/mL)**	1.65 (0.654)	1.58 (0.517)	1.72 (0.617)	1.59 (0.487)	0.28
**NfL (pg/mL)**	19.2 (19.6)	20.5 (28.7)	18.4 (25.3)	15.4 (9.12)	0.12
**MMSE**	28.8 (1.93)	28.7 (2.16)	26.2 (3.52)	25.8 (4.69)	< 0.001

### *APOE4*-race interaction influences AD biomarker levels, showing significant differences in AD pathology

**Fig. 1:**
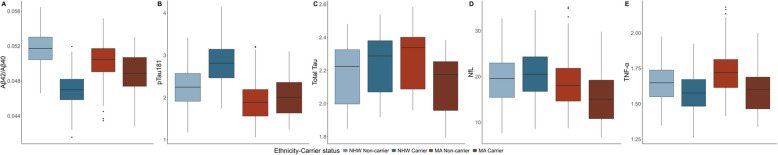
Fitted value boxplots illustrating Alzheimer's disease (AD) pathology biomarkers across four groups defined by APOE4 carrier status and race/ethnicity (non-Hispanic White and Mexican American). Group identities are color-coded and labeled along the x-axis. The plots are intended to visualize patterns consistent with the interaction between APOE4 status and race/ethnicity, as tested in the regression models. While the figure highlights distributional differences across groups, statistical significance reflects the interaction terms derived from linear regression models, not pairwise group comparisons. Significant race/ethnicity × APOE4 interactions were observed for Aβ42/Aβ40 (Panel A, *p* = 0.0215), p-tau181 (Panel B, *p* = 0.001), and total tau (Panel C, *p* = 0.025). No significant interactions were found for NfL (Panel D, *p* = 0.080) or TNF-α (Panel E, *p* = 0.080). All models were adjusted for age, sex, MMSE and years of education

AD-specific group differences in biomarker levels were observed after adjustment for age, gender, and education. There was a significant *APOE*4—race/ethnicity interaction term for Aβ42/Aβ40 (β = 0.0036, 95% CI: (0.0005, 0.0066), *p* = 0.0215), p-tau181 (β = -0.48, 95% CI: (-0.78, -0.18), *p* = 0.001), and total tau (β = -0.240, 95% CI: (-0.450, -0.031), *p* = 0.025) (Fig. 1). No such interactions were observed for NfL (β = -4.720, 95% CI: (-9.998, 0.558), *p* = 0.080), and TNF-α (β = -4.720, 95% CI: (-9.998, 0.558), *p* = 0.080) (Table 2).

**Table 2:** Beta coefficients and 95% confidence intervals are shown for all model terms. FDR-adjusted *p*-values are reported only for the APOE4 × race interaction terms, which represent the primary comparisons of interest

	**Aβ42/Aβ40**	**p-tau181**	**total-tau**	**NfL**	**TNF-α**
Intercept	β = 0.0621 (0.0521, 0.0741)	β = -1.557 (0.1758, 2.1357)	β = 1.79 (1.109, 2.48)	β = -2.105 (-19.379, 15.1686)	β = -2.105 (-19.379, 15.169)
Age	β = -0.0002 (-0.0003,-0.0001)	β = 0.047 (0.040, 0.055)	β = 0.01 (0.001, 0.012)	β = 0.576 (0.442, 0.711)	β = 0.576 (0.442, 0.711)
Gender	β = -0.0008 (-0.0021, 0.0005)	β = -0.217 (-0.34, -0.088)	β = 0.343 (0.252, 0.433)	β = -0.339 (-2.613, 1.936)	β = -0.339 (-2.613, 1.936)
Education, years	β = 0.0001 (0,0.0003)	β = 0.040 (0.021, 0.059)	β = 0.007 (-0.006, 0.021)	β = 0.180 (-0.155, 0.514)	β = 0.180 (-0.1546, 0.514)
MMSE, score	β = 0.0001 (-7.79e-05, 0.0004)	β = -0.093 (-0.1166, -0.0689)	β = -0.017 (-0.033, -9.24e-05)	β = -0.783 (-1.202, -0.363)	β = -0.783 (-1.202, -0.363)
Race	β = -0.0015 (-0.003, 0.0003)	β = -0.0713 (-0.253, 0.1109)	β = 0.108 (-0.020, 0.235)	β = 1.339 (-1.873, 4.550)	β = 1.339 (-1.873, 4.550)
APOE Status	β = -0.0085 (-0.0132, -0.0040)	β = 1.07 (0.62, 1.52)	β = 0.311 (-0.004, 0.627)	β = 6.361 (-1.579, 14.300)	β = 6.361 (-1.579, 14.300)
APOE*Race/ethnicity	β = 0.0036, *p* = 0.0215* (0.0005, 0.0066)	β = -0.48, *p* = 0.001* (-0.78, -0.18)	Β = -0.240, *p* = 0.025* (-0.450, -0.031)	β = -4.720, *p* = 0.080 (-9.998, 0.558)	β = -4.720, *p* = 0.080 (-9.998, 0.558)

* significant at *p*<0.05 for the interaction term

### *APOE4*-biomarker associations vary by race: significant effects in NH white participants but not in Mexican Americans

Two additional sets of analyses were conducted to ascertain whether the group differences were driven by race or *APOE*4. *APOE*4—biomarker associations were tested in race-stratified populations. No *APOE*4 main effects were observed for AD biomarkers, Aβ42/Aβ40, p-tau and NfL among Mexican Americans, only for total tau (β = -0.1650, 95% CI: (-0.3157, -0.0143), *p* = 0.0320) and TNF-α (β = -0.1166, 95% CI: (-0.2223, 0.0109), *p* = 0.0306) (Table 3). In contrast, significant *APOE*4 associations were observed for Aβ42/Aβ40 (β = -0.0049, 95% CI: (-0.0068, -0.0031), *p* = 3.37e-07), p-tau (β = 0.5834, 95% CI: (-0.3755, 0.7913), *p* = 4.92e-08) but not total tau, NfL, and TNF-α, among NH White participants.

**Table 3:** Regression results stratified by race. APOE status term tests whether participants’ biomarker levels differ based on their genetic profile

	**Aβ42/Aβ40**	**p-tau181**	**total-tau**	**NfL**	**TNF-α**
**Mexican Americans Only**					
** Intercept**	β = 0.0623 (0.0511, 0.0736)	β = 0.4997 (-0.4520, 1.4513)	β = 1.2215 (0.5225, 1.9206)	β = -12.2812 (-31.0883, 6.5258)	β = 0.5978 (0.1075, 1.0880)
** Age**	β = -0.0002 (-0.0003, -8.9856e-05)	β = 0.0416 (0.0314, 0.0518)	β = 0.0156 (0.0081, 0.0231)	β = 0.6946 (0.4938, 0.8954)	β = 0.0132 (0.0079, 0.0184)
** Gender**	β = -0.0020 (-0.0040, -1.2309e-05)	β = -0.2294 (-0.3952, -0.0635)	β = 0.2688 (0.1469, 0.3906)	β = 0.3758 (-2.9025, 3.6541)	β = -0.0089 (-0.0944,—0.0765)
** Education, years**	β = 0.0001 (-0.0001, 0.0004)	β = 0.0353 (0.0145, 0.0562)	β = 0.0173 (0.0020, 0.0326)	β = 0.1501 (-0.2616, 0.5618)	β = -0.0148 (-0.0255,—-0.0041)
** MMSE**	β = 5.9117e-05 (-0.0003, 0.0004)	β = -0.0555 (-0.0812, -0.0298)	β = -0.0109 (-0.0297, 0.0080)	β = -0.5870 (-1.0946, -0.0794)	β = 0.0166 (-0.0034, 0.0298)
** APOE Status**	β = -0.0014, *p* = 0.2493 (-0.0039, 0.0010)	β = 0.1318, *p* = 0.2076 (-0.0733, 0.3370)	β = -0.1650, *p* = 0.0320 (-0.3157, -0.0143)	β = -2.9718, *p* = 0.1506 (-7.0261, 1.0824)	β = -0.1166, *p* = 0.0306 (-0.2223, -0.0109)
**NH Whites Only**					
**Intercept**	β = 0.0507 (0.0351, 0.0663)	β = 4.8264 (3.0999, 6.5529)	β = 3.7580 (2.5769, 4.9392)	β = 22.4191 (-5.8836, 50.7218)	β = 1.0192 (0.2571, 1.7812)
**Age**	β = -0.0002 (-0.0003, -9.6435e-05)	β = 0.0514 (0.0403, 0.0625)	β = -0.0011 (-0.0087, 0.0066)	β = 0.4645 (0.2820, 0.6469)	β = 0.0176 (0.0127, 0.0225)
**Gender**	β = 0.0001 (-0.0016,—0.0019)	β = -0.1888 (-0.3830, 0.0055)	β = 0.4030 (0.2701, 0.5360)	β = -0.8918 (-4.0768, 2.2933)	β = -0.0503 (-0.1361, 0.0354)
**Education, years**	β = 0.0001 (-0.0002, 0.0005)	β = 0.0087 (-0.0289, 0.0463)	β = -0.0179 (-0.0436, 0.0078)	β = 0.1790 (-0.4370, 0.7951)	β = -0.0257 (-0.0423, -0.0092)
**MMSE**	β = 0.0004 (-6.8802e-07, 0.0009)	β = -0.2145 (-0.2634, -0.1655)	β = -0.0507 (-0.0842, -0.0172)	β = -1.3081 (-2.1109, -0.5054)	β = -0.0057 (-0.0274, 0.0159)
**APOE Status**	β = -0.0049, *p* = 3.3691e-07 (-0.0068, -0.0031)	β = 0.5834, *p* = 4.9214e-08 (0.3755, 0.7913)	β = 0.0613, *p* = 0.3979 (-0.0810, 0.2035)	β = 1.4557, *p* = 0.4021 (-1.9528, 4.8641)	β = -0.0503, *p* = 0.2819 (-0.1421, 0.0414)

Beta values are reported with upper and lower confidence intervals in parentheses. Significance is denoted with asterisk. Adjusted *p* values (among MA participants): 0.2493, 0.2076, 0.0320, 0.1506, 0.0306, (among NH White participants): 3.369e-07, 4.92e-08, 0.3979, 0.4021, 0.2819

To account for potential confounding due to baseline cognitive status, we ran our primary models including age, gender, education, and MMSE scores as a covariate. Detailed results can be found in supplemental Tables 1 and 2.

## Discussion

There is a growing need to determine if *APOE4* status infers the same risk of AD among Mexican Americans as it would for NH Whites. Although some associations observed were modest, these findings provide important preliminary evidence of racial/ethnic differences in the relationship between APOE4 status and AD biomarkers. Given the cross-sectional nature of the data, longitudinal follow-up will be critical to further elucidate these associations over time.

As an initial step, we set out to examine differences between race/ethnicity and *APOE*4 carriers and non-carriers across AD biomarkers, inflammation, amyloid-beta, neurodegeneration and tau. Indeed, significant differences were found among Aβ42/Aβ40 levels that were primarily driven by *APOE*4 status (*p* = 0.03) with the expected result of *APOE*4 carriers having more amyloid pathology as indicated by lower Aβ42/Aβ40 values among both MA and NH-White participants. However, p-tau181 levels showed significant differences primarily driven by race/ethnicity, with NH Whites exhibiting higher levels (Fig. 1). Interestingly, no significant differences in total tau, NfL, or TNF-α levels were observed across groups. This could be due to the fact that while total tau, NfL and TNF-α are used as biomarkers for AD, they are also non-specific to AD and can be indicative of other conditions [20–22], whereas biomarkers such as Aβ42/Aβ40 and p-tau are more closely associated with AD-dementia [23].

These results challenge the assumption of using *APOE*4 as a universal genetic risk factor for AD for all people, demonstrating that canonical AD biomarkers might behave differently depending on race/ethnicity. Previous research has already shown this to be the case among African Americans who had lower concentrations of p-tau181 and total tau reflecting a significant *APOE*4 interaction with race [24]. Additionally, researchers have a shown that NH Whites have higher levels of canonical AD biomarkers, amyloid beta and p-tau181, marking their greater importance among this group [25].

The nuanced impact of *APOE*4 was further evidenced by significant racial/ethnic—*APOE*4 interactions on Aβ42/Aβ40, p-tau181, and total tau. These interactions were absent for inflammatory and axonal injury markers, such as TNF-α and NfL, suggesting that inflammatory pathways may be less influenced by *APOE*4 or race/ethnicity, but additional considerations (i.e. possible sex differences, involvement of environmental stressors, etc.) and research (i.e. examining these relationships longitudinally) is needed to confirm this.

Follow-up analyses revealed that only among NH Whites, *APOE*4 was associated with Aβ42/Aβ40 and p-tau181, aligning with established knowledge of *APOE*4’s role in amyloid and tau pathology [26, 27]. Notably, no such associations were observed in Mexican Americans. Because previous research has shown that the presence of *APOE*4 allele is associated with increased amyloid production [28] and diminished clearance [29] which is then thought to accelerate tau accumulation [26, 30] we must consider that these mechanisms may not be the primary drivers at play for Mexican Americans or other ethnicities. For instance, while not shown in this paper, there is evidence that stressors related to inflammation may be a bigger risk factor for AD among this group. Some research has found that endorsed depression negatively impacts cognitive outcomes and inflammatory plasma biomarkers among Mexican American, specifically *APOE*4 non-carriers [31, 32]. Coincidentally, we did see higher mean values for inflammation among this group, but they did not differ statistically from MA e4 carriers and NH Whites.

Follow-up studies will include a longitudinal biomarker analysis to start to understand the progression of biomarkers overtime among different ethnicities. Understanding whether baseline differences in biomarkers, such as Aβ42/Aβ40 or p-tau, persist or evolve into cognitive impairment or dementia in Mexican Americans will be of great interest. Additionally, the incorporation of neuroinflammatory markers IL-5, -6, -10 and markers measuring vascular health like PCAM and VCAM will be analyzed in the future to help explore whether alternative pathways may be responsible for the increased incidence of AD in Mexican Americans, potentially linked to metabolic or vascular risk factors.

Previously the relationship between *APOE*4 and AD biomarkers had not been truly tested among Mexican Americans and underscore the importance of studying diverse populations. The findings show genetic and biomarker associations derived from NH Whites may not generalize to underrepresented groups like Mexican Americans adding to a growing number of research that has also shown *APOE*4 is a greater risk factor for AD among NH Whites and not Mexican Americans [11, 12, 33].

### Limitations

Our study has several limitations. First, race and ethnicity were based on self-report, which may not accurately capture underlying genetic ancestry. Additionally, we dichotomized APOE genotype into ε4 carriers (3/4 or 4/4) and non-carriers (2/2, 2/3, or 3/3), which limits our ability to assess potential dose–response effects (e.g., differences between 3/4 and 4/4). This decision was made to ensure sufficient statistical power, particularly within the Mexican American group, where ε4 allele frequency is relatively low. Future studies with larger and more diverse samples will allow for more granular analyses of APOE genotype effects. We also acknowledge that the MMSE, while widely used to assess global cognitive function, may lack sensitivity to subtle or domain-specific impairments. Future work will incorporate more comprehensive cognitive assessments—including measures of memory, executive function, and language—to better characterize cognitive profiles across groups since our study was focused specifically on differences in AD biomarker expression by race/ethnicity and APOE4 status, independent of cognitive performance. Furthermore, as our analyses were based on baseline data, we cannot rule out the possibility that elevated AD-like pathology (e.g., p-tau) may emerge in APOE ε4 non-carriers at later time points.

Our results highlight the importance of continued investigation into racial/ethnic disparities in AD pathology and provide foundational data to inform future longitudinal studies. Finally, while our results suggest that Mexican Americans may follow a distinct pathway to dementia, the underlying mechanisms remain hypothetical. Additional confounding factors—such as lifestyle, socioeconomic status, and comorbidities—that may influence inflammation and AD pathology will need to be carefully explored in future studies.

## Conclusions

These findings provide new hypotheses about the mechanisms underlying AD pathology in Mexican Americans, particularly regarding the role of *APOE*4 and its interplay with race/ethnicity. They highlight the potential for ethnic differences in pathways to AD, suggesting that Mexican Americans may experience dementia through mechanisms distinct from those in NH Whites. These insights contribute to understanding the complex interactions between genetics, ΑD biomarkers, and ethnicity, ultimately aiding in the development of more tailored preventive strategies and early diagnostic tools for populations with a higher incidence of AD.

## Supplementary Information

Supplementary Material 1.

## Data Availability

No datasets were generated or analysed during the current study.
