# Supplementary material for: The effect of APOE4 on Alzheimer’s plasma biomarkers among Mexican Americans in the HABS-HD cohort
Source: Alzheimers Res Ther. 2025 Sep 30;17:208. doi: 10.1186/s13195-025-01845-0 (PMC12482669; doi:10.1186/s13195-025-01845-0)
Supplement: Supplementary file 1 — Supplementary Material 1. [file 13195_2025_1845_MOESM1_ESM.docx]

**Supplemental Material**

*Supplemental Table 1: Regression Results including MMSE as covariate, APOE4—Race interaction term shows whether biomarker levels differ by participants’ racial—genetic profile.*

|  | |  | |  |  |  |  |
| --- | --- | --- | --- | --- | --- | --- | --- |
|  | **Aβ42/Aβ40** | | **p-tau181** | | **total-tau** | **NfL** | **TNF-α** |
| **Intercept** | β=0.0621, *p=*8.67e-33 (0.0521, 0.0741) | | β=-1.557, *p=*0.0208  (0.1758, 2.1357) | | β=1.79, p= 3.23E-07 (1.109, 2.48) | β=-2.105, p= 0.811 (-19.379, 15.1686) | β=-2.105, p= 0.811 (-19.379, 15.169) |
| **Age** | β=-0.0002, *p=*1.84e-07  (-0.0003,-0.0001) | | β=0.047, *p=*1.959e-33 (0.040, 0.055) | | β= 0.01, p= 0.02  (0.001, 0.012) | β= 0.576, p= 9.539E-17 (0.442, 0.711) | β= 0.576, p= 9.539E-17 (0.442, 0.711) |
| **Gender** | β=-0.0008, *p=*0.1984  (-0.0021, 0.0005) | | β=-0.217, *p=*0.0009  (-0.34, -0.088) | | β= 0.343, p= 1.623e-13 (0.252, 0.433) | β= -0.339, p= 0.770 (-2.613, 1.936) | β= -0.339, p= 0.770 (-2.613, 1.936) |
| **Education, years** | β=0.0001, *p=*0.3861 (0,0.0003) | | β=0.040, *p=*3.446e-05  (0.021, 0.059) | | β= 0.007, p= 0.276  (-0.006, 0.021) | β= 0.180, p= 0.180 (-0.155, 0.514) | β= 0.180, p= 0.292 (-0.1546, 0.514) |
| **MMSE, score** | β=0.0001, *p=*0.1834  (-7.79e-05, 0.0004) | | β=-0.093, *p=*3.765e-14  (-0.1166, -0.0689) | | β= -0.017, p= 0.049  (-0.033, -9.235e-05) | β= -0.783, p= 0.0002 (-1.202, -0.363) | β= -0.783, p= 0.0002 (-1.202, -0.363) |
| **Race** | β=-0.0015, *p=*0.1039  (-0.003, 0.0003) | | β=-0.0713, *p=0.442*  (-0.253, 0.1109) | | β= 0.108, p= 0.097 (-0.020, 0.235) | β= 1.339, p= 0.414 (-1.873, 4.550) | β= 1.339, p= 0.414 (-1.873, 4.550) |
| **APOE Status** | β=-0.0085, *p=*0.0002  (-0.0132, -0.0040) | | β=1.07, *p=3.19E-06* (0.62, 1.52) | | β= 0.311, p= 0.053 (-0.004, 0.627) | β= 6.361, p= 0.116 (-1.579, 14.300) | β= 6.361, p= 0.116 (-1.579, 14.300) |
| **APOE*Race/ethnicity** | β=0.0036, *p*= 0.0215*  (0.0005, 0.0066) | | β=-0.48, *p*=0.001*  (-0.78, -0.18) | | Β= -0.240, *p*= 0.025*  (-0.450, -0.031) | β= -4.720, *p*= 0.080  (-9.998, 0.558) | β= -4.720, *p*= 0.080  (-9.998, 0.558) |

Beta coefficients and 95% confidence intervals are shown for all model terms. FDR-adjusted p-values are reported.

*Supplemental Table 2: Regression results stratified by race with the inclusion of MMSE as a covariate. APOE Status term tests whether participants’ biomarker levels differ based on their genetic profile*

| **Mexican Americans Only** | | | | | |
| --- | --- | --- | --- | --- | --- |
|  | **Aβ42/Aβ40** | **p-tau181** | **total-tau** | **NfL** | **TNF-α** |
| **Intercept** | β= 0.0623, p= 1.5122e-25 (0.0511, 0.0736) | β= 0.4997, p= 0.3030 (-0.4520, 1.4513) | β= 1.2215, p= 0.0006 (0.5225, 1.9206) | β= -12.2812, p= 0.2003 (-31.0883, 6.5258) | β= 0.5978, p= 0.0169 (0.1075, 1.0880) |
| **Age** | β= -0.0002, p= 0.0006 (-0.0003, -8.9856e-05) | β= 0.0416, p= 3.4217e-15 (0.0314, 0.0518) | β= 0.0156, p= 4.5356e-05 (0.0081, 0.0231) | β= 0.6946, p= 2.1940e-11 (0.4938, 0.8954) | β= 0.0132, p= 9.8917e-07 (0.0079, 0.0184) |
| **Gender** | β= -0.0020, p= 0.0486 (-0.0040, -1.2309e-05) | β= -0.2294, p= 0.0068 (-0.3952, -0.0635) | β= 0.2688, p= 1.6882e-05 (0.1469, 0.3906) | β= 0.3758, p= 0.8220 (-2.9025, 3.6541) | β= -0.0089, p= 0.8374 (-0.0944, - 0.0765) |
| **Education, years** | β= 0.0001, p= 0.3603 (-0.0001, 0.0004) | β= 0.0353, p= 0.0009 (0.0145, 0.0562) | β= 0.0173, p= 0.0266 (0.0020, 0.0326) | β= 0.1501, p= 0.4743 (-0.2616, 0.5618) | β= -0.0148, p= 0.0070 (-0.0255, - -0.0041) |
| **MMSE** | β= 5.9117e-05, p= 0.7040 -0.0003, 0.0004) | β= -0.0555, p= 2.4738e-05 (-0.0812, -0.0298) | β= -0.0109, p= 0.2585 (-0.0297, 0.0080) | β= -0.5870, p= 0.0235 (-1.0946, -0.0794) | β= 0.0166, p= 0.0141 (-0.0034, 0.0298) |
| **APOE Status** | β= -0.0014, p= 0.2493 (-0.0039, 0.0010) | β= 0.1318, p= 0.2076 (-0.0733, 0.3370) | β= -0.1650, p= 0.0320 (-0.3157, -0.0143) | β= -2.9718, p= 0.1506 (-7.0261, 1.0824) | β= -0.1166, p= 0.0306 (-0.2223, -0.0109) |
| **NH Whites Only** | | | | | |
| **Intercept** | β= 0.0507, p= 3.0914e-10 (0.0351, 0.0663) | β= 4.8264, p= 5.5120e-08 (3.0999, 6.5529) | β= 3.7580, p= 6.9305e-10 (2.5769, 4.9392) | β= 22.4191, p= 0.1204 (-5.8836, 50.7218) | β= 1.0192, p= 0.0088 (0.2571, 1.7812) |
| **Age** | β= -0.0002, p= 0.0001 (-0.0003, -9.6435e-05) | β= 0.0514, p= 9.7440e-19 (0.0403, 0.0625) | β= -0.0011, p= 0.7870 (-0.0087, 0.0066) | β= 0.4645, p= 0.0000 (0.2820, 0.6469) | β= 0.0176, p= 0.0000 (0.0127, 0.0225) |
| **Gender** | β= 0.0001, p= 0.8791 (-0.0016, - 0.0019) | β= -0.1888, p= 0.0568 (-0.3830, 0.0055) | β= 0.4030, p= 3.9978e-09 (0.2701, 0.5360) | β= -0.8918, p= 0.5827 (-4.0768, 2.2933) | β= -0.0503, p= 0.2495 (-0.1361, 0.0354) |
| **Education, years** | β= 0.0001, p= 0.4825 (-0.0002, 0.0005) | β= 0.0087, p= 0.6489 (-0.0289, 0.0463) | β= -0.0179, p= 0.1730 (-0.0436, 0.0078) | β= 0.1790, p= 0.5685 (-0.4370, 0.7951) | β= -0.0257, p= 0.0024 (-0.0423, -0.0092) |
| **MMSE** | β= 0.0004, p= 0.0504 (-6.8802e-07, 0.0009) | β= -0.2145, p= 4.4328e-17 (-0.2634, -0.1655) | β= -0.0507, p= 0.0031 (-0.0842, -0.0172) | β= -1.3081, p= 0.0014 (-2.1109, -0.5054) | β= -0.0057, p= 0.6017 (-0.0274, 0.0159) |
| **APOE Status** | β= -0.0049, p= 3.3691e-07 (-0.0068, -0.0031) | β= 0.5834, p= 4.9214e-08 (0.3755, 0.7913) | β= 0.0613, p= 0.3979 (-0.0810, 0.2035) | β= 1.4557, p= 0.4021 (-1.9528, 4.8641) | β= -0.0503, p= 0.2819 (-0.1421, 0.0414) |

Beta values are reported with upper and lower confidence intervals in parentheses. Adjusted p values are reported for each covariate and biomarker.

**Supplemental Figure 1: Flowchart clarifying participant inclusion.** This flowchart outlines the selection process for participants included in the present study. Starting from the full HABS-HD cohort, individuals were included if they had available plasma inflammatory biomarker data, demographic data, and APOE genotype information. This resulted in a final analytic sample of 792 Mexican American and 785 Non-Hispanic White participants.


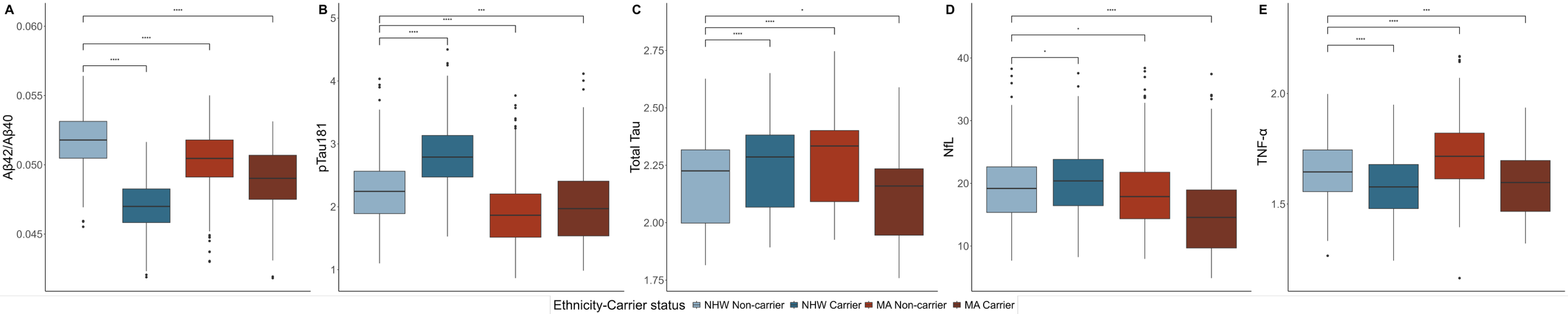


**Supplemental Figure 2: Group-wise comparisons of AD pathology biomarkers using Tukey’s HSD post hoc test.** Boxplots display group-wise comparisons of Alzheimer's disease (AD) pathology biomarkers across four groups defined by APOE4 carrier status and race/ethnicity (non-Hispanic White and Mexican American). Significance bars reflect results from Tukey’s Honestly Significant Difference (HSD) test, using non-Hispanic White APOE4 non-carriers as the reference group. Significance levels are indicated as follows: p < 0.05 (*), p < 0.01 (**), p < 0.001 (***), p < 0.0001 (****).

All comparisons were adjusted for age, sex, and years of education. This supplemental figure is provided to aid interpretation of between-group differences in addition to the interaction effects shown in the main figure**.**
